# 急性髓系白血病异基因造血干细胞移植后阿萨希毛孢子菌感染2例报告及文献复习

**DOI:** 10.3760/cma.j.cn121090-20240319-00101

**Published:** 2024-09

**Authors:** 玮 赵, 曼 陈, 艳丽 赵

**Affiliations:** 1 北京陆道培医院移植科，北京 100176 Department of Transplantation, Beijing Ludaopei Hospital, Beijing 100176, China; 2 河北燕达陆道培医院检验科，廊坊 065201 Department of Laboratory Medicine, Hebei Yanda Ludaopei Hospital, Langfang 065201, China

## Abstract

由于造血干细胞移植后患者免疫力低下，不常见或罕见的酵母菌感染呈上升趋势。对由这些病原体引起的复杂、多学科的感染管理采取恰当的治疗对于优化患者预后至关重要。本文报告了2例血液病患者合并罕见酵母菌阿萨希毛孢子菌感染的诊疗过程，提示我们应及时进行多脏器筛查有无全身播散性感染，早治疗及联合用药可改善预后。

## Abstract (en)

Rare yeast infections have been slowly increasing, given the increasing numbers of patients who are immunocompromised after hematopoietic stem cell transplantation. A considered approach to the complex, multidisciplinary management of infections that are caused by these pathogens is essential to optimize patient outcomes. We reported the management of two patients with combined rare yeast Trichosporon asahii infections, that suggested we should perform timely multivisceral screening for systemic dissemination of infection, and early treatment and combination of medications may improve prognosis.

侵袭性真菌病（invasive fungal disease, IFD）是异基因造血干细胞移植（allo-HSCT）后的常见并发症，主要致病菌为念珠菌和曲霉菌[1]–[2]。近年来，罕见酵母菌感染发病率逐渐增加。本文报告2例合并阿萨希毛孢子菌感染血液病患者的诊治过程并复习相关文献。

## 病例资料

例1，男，14岁，急性髓系白血病M_2_。第2次完全缓解（CR_2_）状态下行同胞全相合造血干细胞移植。移植后4个月血液学复发，化疗联合供者淋巴细胞输注获得CR_3_，后行二次移植（父供子单倍体造血干细胞移植）。预处理分别为改良白消安（Bu）/环磷酰胺（Cy）方案、移植后环磷酰胺（PTCy）方案，GVHD预防采用环孢素A（CsA）+霉酚酸酯（MMF）+短疗程甲氨蝶呤（MTX）。移植过程中应用泊沙康唑预防真菌感染，血药浓度0.5～1.0 mg/L。+1 d患者出现发热，无不适主诉，查体未见阳性体征。血常规ANC 0×10^9^/L，C反应蛋白（CRP）>200 mg/L（参考值0～6 mg/L），降钙素原（PCT）2.22 µg/L（参考值<0.1 µg/L），G试验192 ng/L（参考值<60 ng/L），血培养及血清二代测序（NGS）示阿萨希毛孢子菌（序列数6046），换用两性霉素B脂质体治疗。患者体温无控制趋势，CRP持续>200 mg/L，最高PCT 34.42 µg/L，G试验>600 ng/L。+8 d颅脑CT未见异常，肺部CT示两肺多发斑点状、结节状高密度影。+14 d血培养示阿萨希毛孢子菌，+15 d因肾功能损害停用两性霉素B脂质体。+33 d因感染性休克死亡。

例2，男，26岁，急性髓系白血病M_6_。CR_2_状态下行同胞全相合造血干细胞移植。预处理为改良Bu/Cy方案，GVHD预防采用CsA+MMF+短疗程MTX。单个核细胞输注量为7.2×10^8^/kg，CD34^+^细胞输注量为4×10^6^/kg。因移植前肺感染泊沙康唑治疗有效，移植过程中延用，血药浓度0.5～1.0 mg/L。+6 d患者出现左侧肩部疼痛伴左肩关节活动受限。查体疼痛部位皮肤组织肿胀，边界不清，皮温正常，质软，轻压痛，无皮下结节。血管超声显示贵要静脉近心段及腋静脉内分别见2.4 cm×0.3 cm、0.8 cm×0.2 cm不规则低回声区。予以患肢制动，外周血和中心静脉置管血培养。+8 d患者最高体温38.6 °C，左上肢疼痛加重且延扩展至腕关节，伴尿频、尿急、尿痛膀胱刺激症状。查体阴茎多发斑片状溃疡，左上肢近肩膀及肘窝、睾丸多发痛性结节。血常规ANC 0×10^9^/L，CRP 114 mg/L，PCT 0.7 µg/L，G试验104 ng/L，尿液BK病毒2.0×10^8^拷贝数/ml。超声显示贵要静脉近心段见2.7 cm×0.3 cm不规则低回声区；左上肢疼痛处局部肌肉组织增厚约0.91 cm，其内回声不均匀减低，左上肢近肩膀、左上肢近肘窝处肌层分别见1.6 cm×0.8 cm、1.8 cm×1.0 cm边界清楚低回声区，不除外炎性病变。+6 d血培养阴性，+8 d血培养、尿培养、阴茎分泌物培养及血清NGS均提示阿萨希毛孢子菌感染。临床诊断为“播散性阿萨希毛孢子菌感染（真菌血症、皮肤）”。立即予以拔除中心静脉导管，改用伏立康唑抗真菌治疗（血药浓度1.5～2.5 mg/L），阴茎处溃疡乙醇消毒后以黄连素及两性霉素B湿敷，G-CSF促进造血重建。+13 d患者体温恢复正常，左侧肢体疼痛减轻，溃疡面缩小，左上肢近肩膀及肘窝、睾丸处痛性结节减少。CRP、PCT、G试验结果好转，+15 d白细胞及中性粒细胞植入。心脏超声、颅脑CT未见异常；CT示双肺新发结节状高密度影，肝脏、脾脏、肾脏多发小结节样低密度影，增强后未见明显强化，考虑感染性病变（图1）。修正诊断为“播散性阿萨希毛孢子菌感染（真菌血症、皮肤、肺、肝脏、脾脏、肾脏）”。+20 d外周血培养阴性，尿培养仍见阿萨希毛孢子菌。+22 d加用两性霉素B 25 mg/d治疗，+29 d尿培养阴性。+60 d复查CT示肺、肝脏、脾脏、肾脏多发小结节样低密度影减少、缩小。+84 d患者出现右眼视力下降，视野缺损，左眼视物正常。查体视力右眼光感，左眼0.5，眼压均为10 mmHg（1 mmHg＝0.133 kPa），散瞳眼底可见黄斑下及眼底视网膜出血，黄斑颞侧不规则感染性病灶，房水NGS检出阿萨希毛孢子菌。再次修正诊断为“播散性阿萨希毛孢子菌感染（真菌血症、皮肤、肺、肝脏、脾脏、肾脏、眼睛）”。继续口服伏立康唑联合玻璃体腔内注入伏立康唑+两性霉素B，6次后眼底出血性病灶好转，于移植后6个月行右眼伴有增生膜视网膜脱离复位+经结膜微创玻璃体切除+玻璃体腔穿刺+全视网膜激光光凝术，期间维持血清、房水血药浓度1.5～2.5 mg/L。随访至移植后18个月，原发疾病持续CR，未观察到阿萨希毛孢子菌活动性感染，继续口服伏立康唑治疗。

**图1:**
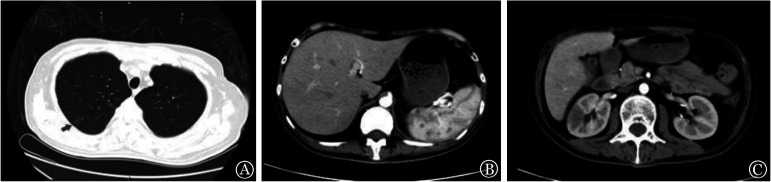
急性髓系白血病患者造血干细胞移植后20 d合并阿萨希毛孢子菌感染CT图像 **A** 双肺可见新发散在多个小结节状高密度影；**B、C** 肝脾脏体积增大，其内可见多发小结节样低密度影，双肾内可见小结节样、肿块样低密度影；增强后均未见明显强化

## 讨论及文献复习

阿萨希毛孢子菌是一种酵母样机会性致病真菌，粒细胞缺乏或免疫缺陷、应用广谱抗生素或糖皮质激素、植入侵入式医疗设备、不恰当应用抗真菌药物是发生严重侵入性毛孢子虫感染的最常见原因（占68％～90％），其中导管相关真菌血症高达80％，死亡率40％～90％[3]–[8]。阿萨希毛孢子菌株可以在植入装置表面形成复杂的三维网格状生物膜，同时内含ERG11基因可降低唑类亲和性，是其高耐药性和死亡率的原因。而控制基础疾病及感染来源、切除植入设备、粒细胞恢复、伏立康唑治疗可改善预后[3]–[5],[8]–[10]。本文2例患者诊断为急性髓系白血病，发病时处于allo-HSCT后粒细胞缺乏期，临床表现为真菌血症、皮肤、肺肝脾肾脏、眼睛多脏器受累，与文献[3]–[10]报道相似。这是目前阿萨希毛孢子菌感染相关报道中同时受累脏器最多的少见患者，提示我们出现真菌血症时，应及时进行多脏器筛查有无全身播散性感染。

在出现阿萨希毛孢子菌播散性感染的血液科病房走廊空气、层流床、设备洁净室、洗脸盆和患者污染物中均发现菌株，提示存在交叉感染可能[11]–[13]。但日本一项最近研究发现健康受试者粪便中阿萨希毛孢子菌定植率为60％，基因型与临床报道分离株几乎相同，推测菌株早期在胃肠道定植可能与后期毛孢子菌病发展有关[8]。Kurakado等[3]在5例COVID-19合并阿萨希毛孢子菌感染患者中分离到4种基因型。阿萨希毛孢子菌优势分子基因型大多为1型，其次为3型和5型，但菌株基因型与毒力、抗真菌药物敏感性及临床预后之间无显著联系[4],[14]–[17]。在2021年版罕见酵母菌感染诊断和管理全球指南[18]中，三唑类药物伏立康唑或泊沙康唑因抗菌活性最高作为一线用药推荐，氟康唑表现出中度敏感性，次之。两性霉素B表现出可变的最低抑菌浓度（MIC），为二线用药推荐。由于阿萨希毛孢子菌对棘白菌素类的天然耐药性，指南并未推荐[3]–[7],[15]–[18]。由于既往报道病例中未提及抗菌药物血清浓度，故其是否在突破性感染发展中充当重要因素尚不清楚。文中患者在泊沙康唑血药浓度达标预防推荐剂量（≥0.7 mg/L）情况下仍出现播散性阿萨希毛孢子菌感染，换用伏立康唑治疗有效，推测后者对真菌14-α-去甲基酶亲和力更高，同时可抑制酵母菌和丝状真菌的24-亚甲基二氢羊毛甾醇去甲基化，最终达到治疗目的，可能免疫功能低下患者更依赖真菌药物的杀菌活性。考虑到伏立康唑不通过肾脏代谢，而两性霉素B具有肾脏高代谢的药代动力学，在患者合并阿萨希毛孢子菌泌尿系感染时，我们尝试了联合用药，获得显著疗效。此外，真菌性眼内炎起病隐匿，治疗周期长，难度大，且易留有脏器功能性永久损伤，抗感染过程中应定期监测眼底。最后乙醇消毒能有效地抑制阿萨希毛孢子菌生物膜形成[10]，小檗碱与两性霉素B联用对阿萨希毛孢子菌浮游细胞及生物膜具有协同破坏作用[19]，治疗患者皮肤溃疡取得了良好效果。

总之，播散性阿萨希毛孢子菌感染是一种罕见酵母菌感染，常发生多脏器受累，早发现早治疗及联合用药可改善预后。
